# Small bowel perforation by a piece of china with a synchronous asymptomatic sigmoid carcinoma: A case report

**DOI:** 10.1186/1757-1626-1-190

**Published:** 2008-09-30

**Authors:** Quentin M Nunes, James J Brousil, Alex Hotouras, Abed M Zaitoun, Magdi Shehata

**Affiliations:** 1 Section of Surgery, Nottingham City Hospital Nottingham, UK; 2 Department of Histopathology, Queen's Medical Centre, Nottingham, UK

## Abstract

A 75 year old gentleman who presented with an incarcerated paraumibilical hernia was found intraoperatively to have small bowel perforation due to a piece of china with a synchronous asymptomatic sigmoid carcinoma.

## Case

A 75-year-old man presented with sudden onset central abdominal pain described as gripping in nature and lasting for approximately 1 hour. The pain subsequently settled to a constant dull ache. He had a regular bowel habit and was otherwise fit and well. On examination the patient had a pulse rate of 94/min, a respiratory rate of 14/min, was afebrile and normotensive. An abdominal examination revealed a paraumbilical hernia approximately 1.5 cm in size, which was irreducible and tender. The rest of the abdomen felt soft with no masses. Bowel sounds were normal and there were no signs of peritonism. A digital rectal examination revealed soft stool. The initial blood tests revealed a raised white cell count of 14 × 10 ^9^/L (normal range 4–11 × 10 ^9^/L). There was no significant abnormality on the abdominal radiograph 1 and the erect chest film showed no evidence of a perforation 2. A presumptive diagnosis of an incarcerated paraumbilical hernia was made and it was decided to operate on the patient. There was a 1.5 cm hernial defect and opening the sac discharged purulent fluid. The abdominal incision was extended. There was a pinpoint antemesenteric perforation in the small bowel approximately 70 cms from the ileocaecal junction with a localised purulent collection. Further examination of the small bowel revealed a broad based, non-inflamed Meckel's diverticulum with a triangular shaped hard object 2 cms proximal to it. This was milked carefully through the proximal bowel and delivered after performing an enterotomy at the site of the perforation. The foreign body was found to be a triangular piece of china, 2 cm at its widest point with sharp edges 3. The enterotomy was sutured. Inspection of the rest of the bowel revealed a circumferential thickening in the sigmoid colon, which was suspected of being a neoplastic lesion. A sigmoid colectomy with a primary anastomosis was performed 4. This was followed by a peritoneal lavage with placement of intraabdominal drains. The patient had an uneventful recovery. The histology of the resected segment revealed a moderately differentiated mucinous adenocarcinoma 5. This was a Duke's B tumour invading the full thickness of the colon with no evidence of vascular or perineural invasion and all ten nodal biopsies proved to be absent of malignant invasion. It is suspected that the foreign body was consumed within a meat pie several days prior to presentation. The patient was of sound mind and has no recollection of consuming the offending object.

**Figure 1:**
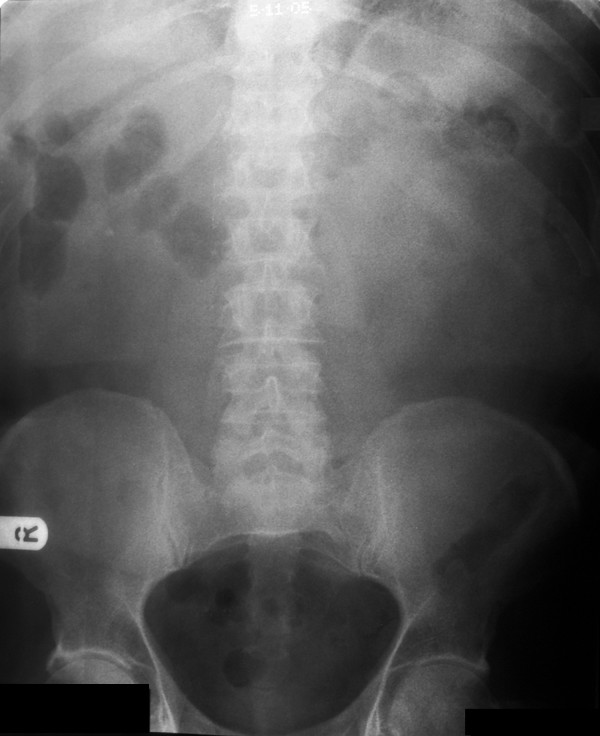
Abdominal radiograph showing no significant abnormality.

**Figure 2:**
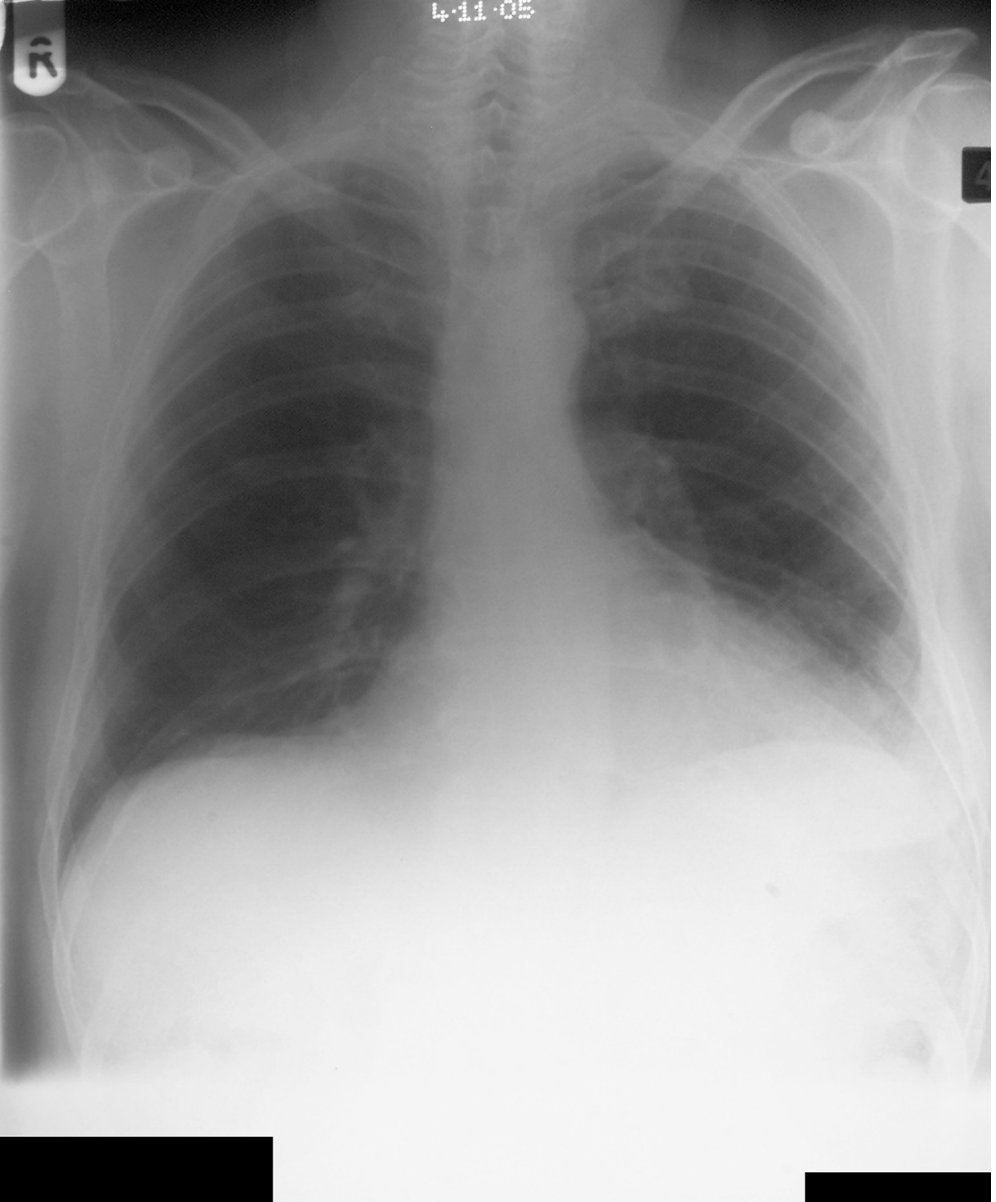
Erect chest radiograph showing no gas under the diaphragm.

**Figure 3:**
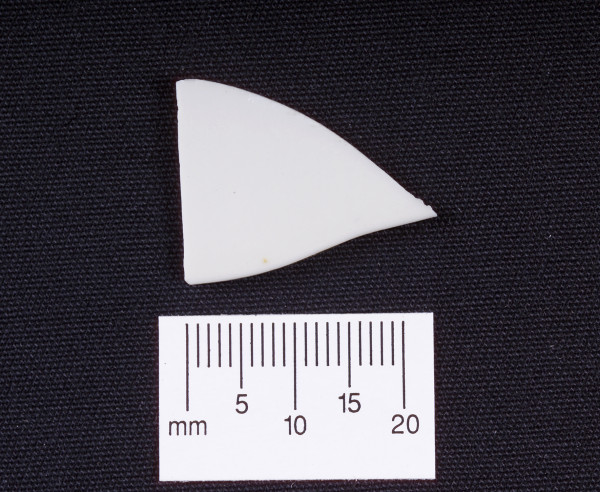
Foreign body – piece of china.

**Figure 4:**
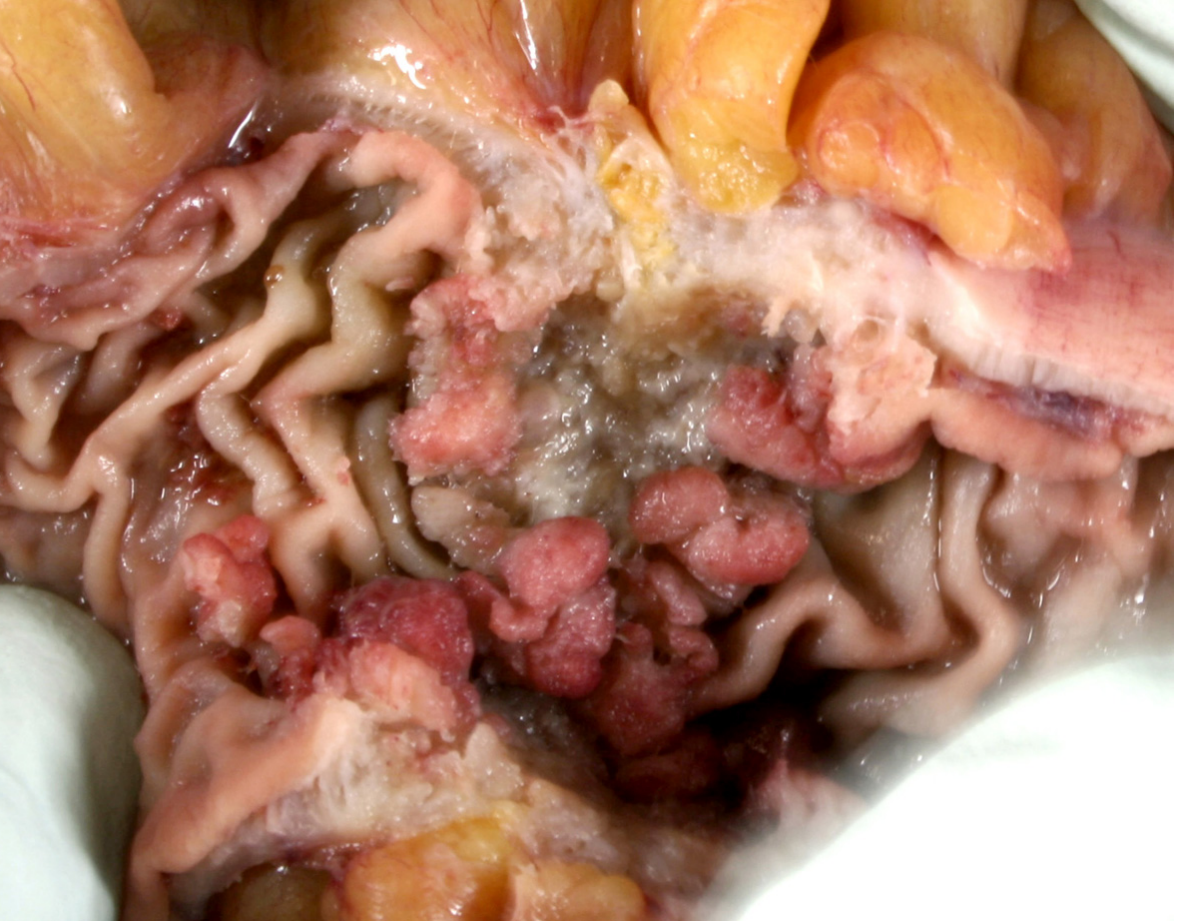
Gross pathology – Sigmoid colon showing circumferential polypoid ulcerated carcinoma.

**Figure 5:**
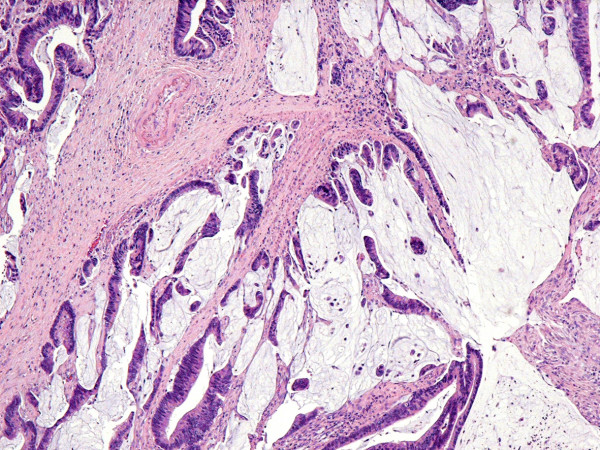
H & E stain × 4 – Moderately differentiated mucinous adenocarcinoma.

## Discussion

Ingestion of a sharp foreign body is mostly accidental, though it may occur in mental impairment and alcoholism. Gastrointestinal perforations can occur at any site along the tract, mainly at narrowings, angulations or in anatomic cul-de-sacs. [1] The ileum is the most common site of foreign body ingestion. [2] A preoperative diagnosis of a perforation by a sharp foreign body is rarely made because the clinical presentation is non-specific and can mimic a number of surgical conditions like appendicitis or diverticulitis. An unenhanced abdominal radiograph shows no specific findings and non-metallic objects are rarely visualised. An erect chest radiograph may show gas under the diaphragm. In our patient, the incarcerated paraumbilical hernia was presumed to be responsible for his clinical presentation necessitating surgery. Foreign body perforation through a sigmoid carcinoma leading to an incidental discovery of the tumour has been described [3,4]. However, small bowel perforation by a piece of china with a synchronous asymptomatic sigmoid carcinoma incidentally discovered at surgery has not been reported. The concurrent aymptomatic Meckel's diverticulum did not require surgery while the asymptomatic Duke's B sigmoid carcinoma was appropriately resected. The occurrence of multiple pathologies in the same patient namely symptomatic incarcerated paraumbilical hernia and the small bowel perforation due to a sharp foreign body along with the asymptomatic Meckel's diverticulum and sigmoid carcinoma make this the first case of its kind to be reported.

## Competing interests

The authors declare that they have no competing interests.

## Authors' contributions

All authors were involved with the concept and design of this report and in the writing of the draft and final versions of the manuscript.

## Consent

Written informed consent was obtained from the patientfor publication of this case report and accompanying images. A copy of this written consent is available for review by the Editor-in Chief of this journal.

## References

[B1] Osler T, Stackhouse CL, Dietz PA, Guiney WB (1985). Perforation of the colon by ingested chicken bone, leading to diagnosis of carcinoma of the sigmoid. Dis Colon Rectum.

[B2] Pinero MA, Fernandez Hernandez JA, Carrasco PM, Riquelme RJ, Parrilla PP (2000). Intestinal perforation by foreign bodies. Eur J Surg.

[B3] Vardaki E, Maniatis V, Chrisikopoulos H, Papadopoulos A, Roussakis A, Kavadias S, Stringaris K (2001). Sigmoid carcinoma incidentally discovered after perforation caused by an ingested chicken bone. AJR Am J Roentgenol.

[B4] Rasheed AA, Deshpande V, Slanetz PJ (2001). Colonic perforation by ingested chicken bone. AJR Am J Roentgenol.

